# DETECTION OF DIARRHEAGENIC *ESCHERICHIA COLI* IN HUMAN DIARRHEIC STOOL AND DRINKING WATER SAMPLES IN OUAGADOUGOU, BURKINA FASO

**DOI:** 10.21010/ajid.v15i1.7

**Published:** 2020-12-14

**Authors:** Isidore Juste Ouindgueta Bonkoungou, Namwin Siourimè Somda, Oumar Traoré, Barthelemy Sibiri Zoma, Zakaria Garba, Koine Maxime Drabo, Nicolas Barro

**Affiliations:** 1 Département de Biochimie-Microbiologie. UFR- Sciences de la vie et de la terre. Université Joseph Ki-Zerbo, 03 BP 7021 Ouagadougou 03, Burkina Faso; 2 Département Technologie Alimentaire (DTA) / IRSAT / CNRST, Burkina Faso, 03 BP 7047 Ouagadougou 03; 3 Unité de Formation et de Recherche en Sciences Appliquées à la Technologie (UFR/SAT). Université de Dédougou, BP 176 Dédougou; 4 Laboratoire National de Santé Publique (LNSP), 09 BP 24 Ouagadougou 09, Burkina Faso; 5 Polygon Bio Services SARL,09 BP 969 Ouagadougou 09, Ouagadougou, Burkina Faso 969; 6 Unité de Recherche Clinique de NANORO, IRSS-CNRST, BP: 218 Ouaga 11 Burkina Faso; 7 Institut de Recherche en Sciences de la santé, CNRST, 03 B.P. 7192 Ouagadougou 03 Burkina Faso

**Keywords:** 16-plex PCR, drinking water, diarrhoeagenic *Escherichia coli*, Burkina Faso

## Abstract

**Background:**

The presence of diarrheagenic *Escherichia coli* (DEC) in drinking water, is a grave public health problem. This study was aimed at characterization of diarrheagenic *Escherichia coli* isolated from drinking water and faecal samples from diarrheic patients in Ouagadougou, Burkina Faso.

**Materials and Methods:**

A total of 242 water samples consisting of 182 potable sachets and 60 from boreholes were collected in the period between October 2018 and April 2019 in the city of Ouagadougou. Faecal samples were also collected from 201 diarrheic patients visiting National Public Health Laboratory for a biological diagnosis by coproculture. The presence of virulence genes associated with DEC was determined by 16-plex polymerase chain reaction from bacteria culture.

**Results:**

From drinking water, we found 17% (42/242) *Escherichia coli* isolates in which 1% (2/242) DEC were detected. Among analyzed samples (182 sachet water versus 60 borehole water), the two DEC (01 ETEC and 01 EPEC) were detected in sachet water. DEC were detected in 20% (40/201) of patients. Enteroaggregative *Escherichia coli* (EAEC) were mostly detected in 10% followed by Enteropathogenic *Escherichia coli* (EPEC) in 4%, Enteroinvasive *Escherichia coli* (EIEC) in 2%, and Shiga toxin-producing *Escherichia coli* (STEC) 0.5%. However, Enterotoxigenic *Escherichia coli* (ETEC) was not detected alone, but in co-infections with EAEC.

**Conclusion:**

The present study documented the prevalence of *Escherichia coli* pathovars associated in patients with diarrhea, and shows that drinking water might be a source of DEC transmission in human.

## Introduction

Diarrheal diseases are among the main causes of morbidity and mortality in Africa and mainly affect children under 5 years old (Adjuik *et al.*, 2006). They are usually caused by at least one bacterial, viral or parasitic agent. These infections are often linked to non-compliance with the conditions of good hygiene practices during foods processing and especially poor quality of drinking water. According to World Health Organization (WHO), better access to drinking water is a key factor in the reduction of diarrheal diseases. However, access to water is poor the cities of sub-Saharan Africa where only 20% of the population is supplied by an unimproved source of water (Aubry and Gaüzère, 2012). Water sources can be investigated for detection of fecal contamination; high fecal levels can mean that water contains pathogens by testing for the presence of Escherichia coli (Cowan, 2018). *Escherichia coli* is a member of the faecal coliform group that is mostly considered as a specific indicator of faecal pollution. They are normally found in the feces of humans or other warm-blooded animals. Most strains of *Escherichia* coli are harmless, and their presence in the water only suggests that faecal contamination may have occurred and that disease-causing organisms may be present (Aubry and Gaüzère, 2012). According to their virulence properties, symptoms of the disease that they cause, species and age group wherethey are found, *Escherichia coli* is classified into: enteropathogenic *E. coli* (EPEC), enterotoxigenic *E. coli* (ETEC), Shiga toxin-producing *E. coli* (STEC), enteroinvasive *E. coli* (EIEC) and enteroaggregative *E. coli* (EAEC) being the most important (Kaper *et al.*, 2004). Presence of DEC in drinking water has been mentioned by previous studies (Hunter, 2003) and all are known to be endemic in essentially all developing countries (Jafari *et al.*, 2012). Its presence is therefore important to assess the prevalence of diarrheal diseases and identify the associated risk factors, in particular, those linked to the consumption of water and sanitation. Over the past decade, researchers have focused studies on *Escherichia coli* pathovars of clinical and environmental origin, which are responsible for diarrhea in children under 5 years old and adults in Burkina Faso (Bonkoungou *et al.*, 2011; Kagambèga *et al.*, 2013; Somda *et al.*, 2017). However, up to date, no study to our knowledge has characterized the *Escherichia coli* pathovars in drinking water, in order to establish the role of drinking water in development of diarrhea diseases in Burkina Faso.

In this study, virulence genes of DEC were detected in drinking water for the first time in Burkina Faso through potable sachets and boreholes water. Also, DEC prevalence were investigated in stools samples from diarrheic patients in the same city (Ouagadougou), Burkina Faso.

## Materials and Methods

### Sample collection

In this study, sampling considered the fact that potable sachets and boreholes water have emerged as an alternative source of drinking water in the capital city, Ouagadougou in Burkina Faso. We included stool samples in order to update DEC prevalence in the area and during the period of drinking water sampling.

The study was conducted at the National Public Health Laboratory (LNSP) in the capital city of Ouagadougou, Burkina Faso. LNSP is a multidisciplinary institute with the following departments: Environment, Food, Drugs and Medical Biology departments.

This study was part of LNSP routine activities in which diarrheic stool samples were collected from patients visiting the Medical Biology department for a biological diagnosis by coproculture prescribed by a health worker as described in our previous study (Somda *et al.*, 2017). Stool samples were collected from October 2018 to April 2019 from 201 patients visiting a health worker due to gastroenteritis. All samples used in this study had been anonymized and made untraceable before storage. The study received permission from the LNSP authorities of Burkina Faso and verbal informed consent was acquired from patients or parents/legal guardian prior to enrollment.

For drinking water, a total of 242 samples from LNSP’s Environment Control Department collected during routine analysis (from October 2018 to April 2019) of drilling water and sachet water were included. Samples of sachet water were taken randomly in their original packaging on the storage site according to LNSP water routine activities sampling plan and sent to the laboratory for analysis. The sampling of drilling (borehole) water was exhaustive, involving all the companies which were the subject of a quality control request. The samples were taken directly at the source in an aseptic manner in sterile bottles, placed in coolers, and sent immediately to the laboratory for analysis.

### Microbiological analysis

***Drinking water samples:*** The membrane filtration method was used: 100 ml of drilling water and 250 ml of sachet water were homogenized and filtered through a cellulose nitrate filtration membrane with a porosity of 0.45 µm and then placed on an Extra Selective Chromocult Agar and incubated at 37° C for 24 hours. After incubation, suspected colonies (dark blue to purple) were scraped with sterile Pasteur pipette and put in sterile cryotubes containing brain-heart infusion broth added 15% glycerol and conserved at -20° C for molecular analysis.

***Clinical samples:*** All collected stool samples were cultured on MacConkey Sorbitol agar (HIMEDIA, M298I-500G, India) and incubated at 37° C for 24 hours. After incubation, bacterial mass was scraped using steriled Pasteur pipette and put in sterile cryotubes containing Cervel Heart Infusion Broth (BICC) added 15% glycerol and conserved at -20° C for *E. coli* pathovars detection.

### 16-plex PCR assay

DEC pathovars Shiga toxin-producing *Escherichia coli*, Enteropathogenic *Escherichia coli*, Enterotoxigenic *Escherichia coli*, Enteroinvasive *Escherichia coli*, and Enteroaggregative *Escherichia coli* (STEC, EPEC, ETEC, EIEC, and EAEC) were detected by using 16-plex PCR technique as described by (Antikainen *et al.*, 2009). Principal genes detected were uidA, pic, bfp, invE, elt, ent, escV, aggR, stx1, stx2, estIa, estIb, and ast (Müller *et al.*, 2007), hlyA (Antikainen *et al.*, 2009), eaeA (Vidal *et al.*, 2005), ipaH (Brandal *et al.*, 2007)using specific primers as previously described (Bonkoungou *et al.*, 2011; Somda *et al.*, 2017).

***Extraction:*** DNA was extracted by thermal shock. For this, suspected colonies stored at -30 °C were transplanted on a MacConkey Sorbitol agar and incubated at 37° C for 18 to 24 hours. After incubation time, with a sterile loop, a bacterial mass was added to 250 μL of sterile distilled water and heated to 100º C for 10 min and centrifuged (11337g for 10 min). The supernatant was then collected in a new sterile 1.5 ml Eppendorf tube and stored at -20 °C for molecular studies.

***Amplification:*** PCR was performed by using optimized ready-to-use PCR master mix (Solis Biodyne) with 16 primers pairs listed above in a single PCR reaction, as described previously (Bonkoungou *et al.*, 2011; Somda *et al.*, 2017). The criteria to determine DEC were as described previously (Bonkoungou *et al.*, 2011; Somda *et al.*, 2017)

***Electrophoresis:*** The five E. coli pathovars were determined by their specific size on 2% agarose gel and visualized under UV light after staining with ethidium bromide. Reference strains and previous studies isolated as used as positive controls strains as described previously (Bonkoungou *et al.*, 2011; Somda *et al.*, 2017). Distilled water was used as negative control. Single PCRs were used to confirm all PCR positive results.

**Data analysis:** All data were stored and analyzed using Excel 2016 software.

## Results

### 

#### Quality of drinking water and and prevalence of DEC

A total of 17% (42/242) *E. coli* isolates were isolated from drinking water among analyzed samples (182 sachet water versus 60 drilling water). Out of 42 *E. coli*, 24 (13%) were from sachet water and 18 (30%) from drilling water.

Of 42 isolates analyzed, 2 (1%) DEC were detected as 1 EPEC and 1 ETEC. All 2 DEC were from water in sachet.

**Figure 1:**
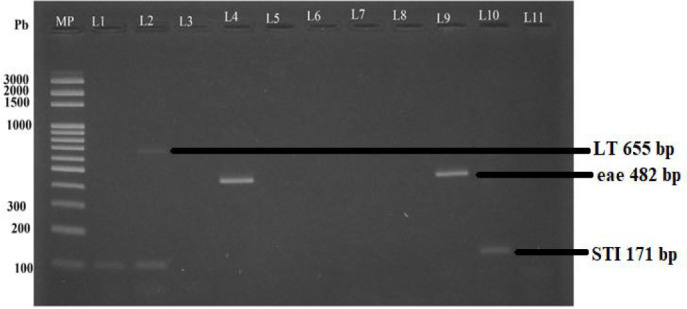
Electrophoresis gel of DEC detection in drinking water **Legend:** MP = ladder, bp = base pairs, L1, L2, L4 = positive control, L3 = negative control, L9 = positive to EPEC (Enteropathogenic *Escherichia coli*); L10 = positive to ETEC (Enterotoxinogenic *Escherichia coli*).

*E. coli* was more prevalent in drilling water than sachet water (30% versus 13%) but no DEC was detected in drilling water **(Figure 1).**

#### Distribution of diarrheal stool samples collected and prevalence of DEC

Out of 201 stools analyzed, 105 samples (52 %) were from male patients. Stratified by age, 90 samples (45 %) were from children under 5 years old, 30 samples (15 %) were from patients aged between 6 and 18 years old, and 81 samples (40%) were from patients over 18 years old.

Out of 201 stool samples analyzed, a total 40 DEC (20%) were detected. All the 5 pathovars were detected either alone or in coinfections. EAEC was the most commonly detected 10 % (21/201) following by EPEC 4% (08/201), EIEC 2 % (05/201) and STEC 1 % (01/201). However, ETEC were not detected alone, but in co-infections with others pathovars. More than one DEC was detected in 5 samples (2%). **(Table 1)**.

**Table 1:** Distribution of the different DECs detected according to age

Pathovars/ages (years)	Ages groups			Total n=201 (%)

	[0 - 5] n=90 (%)	[6 - 18] n=30 (%)	> 19 years n=81 (%)	
EAEC	13 (14)	05 (17)	03 (4)	21 (10)
EPEC	06 (7)	02 (7)	00	08 (4)
EIEC	03 (3)	01 (3)	01 (1)	05 (2)
STEC	01 (1)	00	00	01 (1)
EAEC + EIEC	00	01 (3)	00	01 (1)
EAEC + ETEC	01 (1)	00	00	01 (1)
EAEC + STEC	01 (1)	00	01 (1)	02 (1)
EAEC + EPEC	01 (1)	00	00	01 (1)

TOTAL	26 (29)	09 (30)	05 (6)	40 (20)

**Legend:** EAEC = Enteroaggregative *Escherichia coli*, EIEC = Enteroinvasive *Escherichia coli*, STEC = Shigatoxinogenic *Escherichia coli*, ETEC = Enterotoxinogenic *Escherichia coli*, EPEC= Enteropathogenic *Escherichia coli*, n = number

#### Prevalence of diarrheal Escherichia coli according age and sex

Out of 40 DEC detected, 17% (18/105) were from males and 23% (22/96) were from females. Of the 40 DEC detected 26 (29 %) were from children under 5 years old, 9 (30 %) in patients aged between 6 and 18 years old, and 5 (6%) from patients over age 19 years old. Among the 26 cases detected in children under 5 years, the composition was as follows: EAEC, 14 %; EPEC, 7%; EIEC, 3 %; STEC, 1 %; and 3 co-infections EAEC + ETEC, EAEC+ STEC, EAEC+EPEC at 1 % in each of the cases **(Table 1)**. In patients aged between 6 and 18 years old, EAEC were found in 17% followed by EPEC in 7%, EIEC in 3%, and 3% of 1 co-infection of EAEC+EIEC. Among patients aged over 19 years old, EAEC were detected in 4% followed by EIEC in 1% and 1% of co-infection of EAEC+STEC.

## Discussion

In this present study, *E. coli* pathovars were detected in the first time in drinking water in Burkina Faso.

*Escherichia coli* were isolated in 17% and DEC in 1% of drinking water analyzed. This finding could be because sachet water is generally packaged and stored in unsanitary conditions, which allows the contamination and proliferation of bacteria. The lack of safe drinking water is one of the leading causes of death especially in children under 5 years old (WHO, 2008).

The *E. coli* pathovars detected in drinking (sachet water) were mainly composed of EPEC and ETEC. Previous studied in Egypt (Ahmed *et al.*, 2014), Bangladesh (Talukdar *et al.*, 2013) and Brazil (Lascowski *et al.*, 2013) reported the presence of DEC in drinking water in these countries. In Burkina Faso, packaged water is generally intended for direct consumption since they have a reputation to be potable. Sachet water is the most commonly consumed packaged water due to their low prices which made them more affordable than bottled water.

They are often obtained from drilling water, but sometimes obtained from surface water after treatment. It is supposed to be free of pathogenic germs and therefore suitable for consumption. Nowadays, many sachet water conditioning companies have emerged in Burkina Faso. However, some owners ignore the rules of good manufacturing and hygiene practices. Some are produced directly at home without any recipient of hygiene. All these facts could be at the origin of the contamination of these waters by *Escherichia coli* diarrhea. Others studies shown that water from public standpipes, and wells is collected at the source, carried to, and stored in the household affording multiple opportunities for contamination such that final water quality is often worse than in the associated source (Lascowski *et al.*, 2013; Hunter, 2003)

The DEC prevalence in patients with diarrhea (20%) was lower than that (45%) found in our previous study in 2011 in Burkina Faso (Bonkoungou *et al.*, 2011; Bonkoungou *et al.*, 2013). Overall, the decrease in DEC cases over the years in Burkina Faso might be explained by the fact that population has adopted better hygiene measures since the recent Ebola epidemic in west Africa in 2014. In accordance, other studies carried out elsewhere have shown that these pathogens are indicators of poor compliance to hygiene standards (Gomes *et al.*, 2016).

Of the patients participating in this study, children under the age of 5 years were the most likely to have diarrhea caused by DEC, 65% (26/40) of total DEC. This observation might be explained by the weak immune system in children and supporting the well-documented role of DEC in childhood diarrhea in Burkina Faso (Bonkoungou *et al.*, 2011; Bonkoungou *et al.*, 2013).

In our study, the most frequently detected DEC was EAEC. In recent years, EAEC although specific to travelers’ diarrhea, has mostly been identified as a diarrheal agent causing acute and chronic diarrhea in all age groups and common infection primarily in newborns and immunocompromised patients (Gomes *et al.*, 2016). In other studies, EAEC was associated with diarrhea due to malnutrition in children living in developing countries and in HIV patients (Kaur *et al.*, 2010). Recent studies have shown high prevalence of EAEC in Burkina Faso and elsewhere, for example, in Gambia and United States (Ikumapayi *et al.*, 2017; Imdad *et al.*, 2018; Konaté *et al.*, 2017; Saka *et al.*, 2019; Somda *et al.*, 2017). However, some studies have reported that EAEC is not associated with diarrhea (Bonkoungou *et al.*, 2011; Hien *et al.*, 2008; Keskimäki *et al.*, 2000), and that EAEC was probably endemic among people in local communities and might not be a primary cause of diarrhea.

EPEC was found in 4% and only detected in children ≤ 5 years old. The EPEC prevalence was lower than previous study in Burkina Faso (Bonkoungou *et al.*, 2011; Bonkoungou *et al.*, 2013). It was expected to find EPEC children since it is known that EPEC are the leading cause of infantile diarrhea in developing countries (Trabulsi *et al.*, 2002). The low prevalence of STEC, 2%, is similar to a previous study in Burkina Faso (Bonkoungou *et al.*, 2011).

Among the samples negative for DEC, other pathogens such as viruses, parasites or bacteria, which were not investigated in this study might be responsible for diarrhea.

In Burkina, previous studies have reported the detection of ETEC in grilled chickens, cow dung and organic manure in Burkina Faso (Bako *et al.*, 2017; Kambire *et al.*, 2017; Somda *et al.*, 2018). The similarity of pathovars found in human stool samples, drinking water, and in cow dung in Burkina Faso could be because the aquatic environments are contaminated with animal fecal droppings. The use of new technologies such as Next-generation sequencing will help researchers from developing countries like Burkina Faso to establish routes of transmission for DEC by sequencing *E. coli* strains isolated from various samples in the future.

## Conclusion

This study detected the presence of DEC, particularly ETEC and EPEC, in both diarrheal stool samples and drinking water. These results suggest that routinely consumed water, which should be free of pathogens, is a potential source of DEC contamination in humans with the greatest effect in children under the age of five years. From our findings, drinking water is a potential source of transmission of DEC in Burkina Faso. Therefore, more effort must be made by the public health participants involved in one health approach (environment, human and veterinary medicine) to reduce food and water borne diseases.

**List of Abbreviations:**
DEC: = Diarrheagenic *Escherichia coli*
LNSP: = National Public Health Laboratory
BF: = Burkina Faso
BICC: = Cervel Heart Infusion Broth
EAEC: =Enteroaggregative *Escherichia coli*
EPEC: = Enteropathogenic *Escherichia coli*
ETEC: = Enterotoxigenic *Escherichia coli*
STEC: = Shiga toxin-producing *Escherichia coli*
EIEC: = Enteroinvasive *Escherichia coli*
PCR: = polymerase chain reaction
